# Transactions of the Illinois State Medical Society for the Year 1853

**Published:** 1854-03

**Authors:** Samuel Thompson

**Affiliations:** Albion, Illinois


*Transactions of the Illinois State Medical Society, for the year* 1853. "On the Uses of Turpentine; by Samuel Thompson, M.D., of Albion, Illinois.

*A Dictionary of Practical Medicine*, Comprising General Pathology, the Nature and Treatment of Diseases, Morbid Strictures, &c: by James Copland, M. D., F. R. S. Re-published by Harper & Brothers, New York.

"RENDER UNTO CÆSAR THE THINGS THAT ARE CÆSAR'S."

(*Continued from last Number.*)

Since the preceding part of this article was written, we have received the first No. of the *Retrospect* for the present year. It contains two articles on the use of turpentine in hemorrhagic diseases. Dr. Oke (page 201) recommends turpentine and kino to arrest injurious bleeding from piles. We will extract the first case reported by him. "A tradesman, aged forty-five, the father of a large family, had been for a considerable time subject to daily hemorrhage from the veins of the rectum. He had lost so much blood with every stool, as to become blanched and exhausted. Several medical men had been consulted, who endeavored to restrain the bleeding by a variety of remedies, both internal and external, but without success; and the patient had prepared himself for a fatal issue. Being requested to visit him at this time, I placed him at once under the above treatment. The effect was really marvellous. The bleeding was almost immediately controlled; and, what greatly surprised me, the stools, which had been of an ash color—and they are always of a light color in these cases—assumed a healthy and well digested appearance. It is therefore probable that the turpentine not only acted as a styptic, but also as a deobstruent in removing, in some way or other, the hepatic obstruction. Be this as it may, the bleeding never returned; the patient gradually regained his good looks, and has for many years enjoyed excellent health." He concludes his remarks thus: "Turpentine as a styptic for internal passive hemorrhage stands unrivalled." True, Dr., would that we could say as much for your honor. Dr. Copland recommends turpentine both by the mouth and in enemata in the same disease, and it was published long before your observations, and you knew it, and yet no allusion to him appears on the record. Piles are a very common form of disease in our section of the country and have their origin, we think, partly from the *fine* flour invariably used in making bread, and partly from the *patent pills* almost as invariably used to remove the costiveness produced by the fine flour. During a practice of nine years in England, we never met with a case of piles in the peasantry, who eat coarse wheaten or oaten bread, and take no patent pills.

Mr. Smith (*Retrospect*, same No., page 319,) relates two cases of purpura which came under his treatment the past year—the first was of a chronic character and, under the usual treatment, the amendment was chronic too. The second case was somewhat acute, and the amendment from the use of turpentine, was rapid and complete. "I certainly believe, (he remarks) that in all passive hemorrhages, turpentine will be found valuable in its effects; and in this class of diseases we must generally place purpura," and he acknowledges his obligation to Dr. Copland.

As purpura is not an infrequent disease, we will now give a somewhat lengthy extract from Copland on its treatment, but it will amply compensate the reader for its perusal. "*Viewing the disease as essentially dependent upon impaired vital tone, and cohesion of the capillaries and of the several tissues, with more or less manifest change in the blood, either proceeding from or connected with impaired assimilation and excretion.* I have hardly ever directed vascular depletion, but have prescribed those remedies which appeared to me the best suited for the removal of these pathological states; and I have never found, in the many instances in which I have prescribed it since 1817, the *oleum terebinthina* fail in removing the disease when prescribed in a suitable form or dose, or in such combinations as the peculiar features of the case required. Numerous other means will often succeed in curing this malady; but there is none so efficacious as this in the hemorrhagic states of the disease, and none which will be more beneficial, conjoined with purgatives, in the several circumstances requiring a purgative treatment. If we wish to arrest the hemorrhagic disposition, the turpentine should be given in doses varying from half a drachm to a drachm three or four times daily; and if the vital powers be much depressed, a few drops of the tincture of capsicum, or some aromatic tincture, may be conjoined with it. If it be more desirable to act upon the bowels, then it may be prescribed in much larger doses, with castor oil, on the surface of some aromatic water, or in any other mode; or it may be administered similarly conjoined in enemata. If the exhibition of it by the mouth produce *vomiting*, this occurrence may prove salutary, or may even be promoted, as tending to emulge the biliary ducts, and to remove conjestion of the abdominal viscera. If, on the other hand, it should be preferred neither to risk nor to produce this effect, or even the unpleasant sensation it may produce when thus exhibited, then the administration of it in enemata, in moderate doses, with a few drops of tinctura opii, or with a drachm or two of tinctura comphora comp., repeating the enemata frequently, or according to the period of their retention, and to their action on the bowels, will be very beneficial. If the patient complain of abdominal pains and flatulence, epithems of turpentine, or liniments or embrocations containing it may be applied over the abdomen, or frictions with these may be directed." (*Opus cit. article purpura.*)

On the 12th of last September we saw, in consultation, a case of purpura. We saw the same patient about two weeks previously suffering under a severe attack of dysentery, but was convalescent when the former disease attacked him. Perhaps it would be more correct, to call the case *hemorrhagia universalis*, for there was no portion of mucous membrane, so far as we could ascertain, through which blood did not exude, and no portion of skin fell from either ecchymoses or vibices or petechiæ. He had been judiciously and energetically treated. Tonics, stimulants and styptics amongst which was turpentine had been given as freely as the stomach would bear—the same course was continued but, apparently, without the least effect, for the general hemorrhage continued and the patient succumbed to the disease the day after we saw him. It is possible the turpentine was not given in a "suitable form or dose", nevertheless we are a little inclined to the opinion that had Copland himself treated this case, he would, in the next edition of his Dictionary, record one unfavorable issue. We approach Dysentery, and its treatment with turpentine, with some hesitation and no little diffidence. As far as we have proceeded, the cases requiring the use of Turpentine, have been so clear, that an observant Physician would be able to prescribe it without any or with but little, doubt of its success. But such plain indications for its administration do not exist in dysentery, as in any other of the diseases for which we have prescribed it. It is true that in some stages of the diseases, and in certain conditions of the system, either the effect of or complicating dysentery, we have frequently prescribed it without doubt and without disappointment; but we have repeatedly given it, (see our report in the last transactions of the state society) with complete success, where the most striking indications for its exhibitions were generally wanting; and we were induced to use it in these cases solely from the fact—that the Diarrhœas preceding the Dysentery in the same localities were cured more rapidly by turpentine than by any other remedy or combination of remedies. These cases were treated in 1852. In the autumn of last year we gave turpentine in Dysentery without any benefit, and yet there was no difference in the symptoms, that we could appreciate, except that the disorder as far as we could ascertain, was located higher in the bowels than in the preceding year. There is a good deal about this disease we do not understand or pretend to understand. Since its first appearance in this county in 1851 it has visited us every autumn, and the treatment that was successful in the first year, has been useless, and worse than useless, in every succeeding year. Each visitation of the disease has required a different course of treatment—and such appears to be its character from the earliest records of medicine to the present time.

Dr. Copland says "The terebinthinates are valuable remedies in the asthenic and chronic forms. They were recommended by the the author (Med &. Phys. Journal, Vol. 46, page 107,) and have since been employed by several physicians. They are not contra-indicated in the inflammatory varieties, although bleeding should be premised; and when exhibited so as to act gently on the bowels, or in small enemata, they counteract the tendency to sloughing or ulceration; particularly in the asthenic varieties." There is as much information on this subject in the above extract as we can meet with any where, or we can hope to convey.

We will conclude by giving a short description of one of the worst cases that fell under our care in 1851. The patient, rather past middle age, had mucous and bloody discharges three days before we saw him—he had been taking frequently during those days a composition of his own making—equal parts of Castor Oil, Molasses and Rum—but without any benefit—as there was hepatic derangement we proposed giving him a full dose of Calomel—to this he objected—and he was treated with sedatives, refrigerants, counter-irritation, aperients, &c. for eighteen days—the case all the time apparently tending to a fatal termination. On the fourteenth day of our attendance the right side of the face began to swell and put on an erysipelatous appearance, and we scarified it at once and deeply; on the eighteenth day a considerable abscess had formed a little above the angle of the lower jaw, it was opened and discharged about an ounce of unhealthy pus. By this time the case was almost hopeless, the patient's strength well nigh exhausted, and the discharges from the bowels almost continuous and showing a bad condition of the disease. During this visit we were somewhat surprised by another physician making his appearance, and as no intimation had been given of any dissatisfaction with us—without saying a word we withdrew from the case. (The Dr. was not orthodox.) Four days afterward, we were sent for again and the case had evidently been unfavorably progressing during the time. The first question that we asked ourselves was, "Is it of any use to give anything? and if so what?" We concluded that if any remedy would change the morbid condition of the color—turpentine would—if it would be thrown sufficiently high to reach the disease. Four ounces of turpentine mixed with the same quantity of mucilage were thrown as forcibly as it could be into the bowels; he instantly complained of excruciating pain and wished to evacuate the injection—but we prevented it by firmly grasping the sphincter and kept it retained for about a minute; he then passed it—but he complained of so much distress, comparing it to hot coals in his fundament—that we washed out the syringe and injected half a pint of cream with considerable relief. We then retired to another room and took dinner; on our return the countenance had brightened so much that we gave cautiously a somewhat favorable prognosis. In our anxiety to act promptly and energetically, we had entirely overlooked the excoriating effect of long-continued irritating discharges—or we could have partly prevented the smarting from the injection by anointing the anus and contiguous parts. The next discharge from the bowels passed in about an hour ; it contained some fœcal matter, a little mucous, and blood of a vermillion tint—from this time the improvement was rapid and in three days every vestige of the disease had disappeared. This is another case where the beneficial effect of the remedy on disease was immediately felt by the system and manifested through the countenance.

Dr. Copland recommends turpentine for the removal of Colic in almost all its varieties, and in lead Colic he gives it in large doses combined with Castor Oil. It is frequently prescribed or rather given by mothers in this county for the same disease ; and there can be no doubt, from its popularity, that it is generally efficacious.

We will conclude our remarks on Colic by relating a case that has some very singular and interesting features.

Some eight years ago we were consulted by a middle aged man —he was low spirited and desponding, but had little to complain of beyond irregularity in his alvine discharges, accompanied more by a general uneasiness than actual pain in the bowels. On the most careful examination of his case, there was not anything very tangible about it, except we could perceive some degree of hardness in the left side of the umbilicus, and which we at the time thought was situated on the transvere arch of the colon. After treating the case mildly for a few weeks, we presented him before the old Peoria District Medical Society, and a variety of opinions was given as to the cause of his sickness—but, like the case itself, there was nothing very tangible in them. The case remaining too obscure for specific practice, we treated him with tonics and aperients to which a little blue mass was added, and so it progressed without any material change for some weeks, and not knowing really what to do, we gradually lengthened the intervals between our visits. The patient during this time was generally able to travel about the country. One morning we were sent for in haste, and on arriving found, that during the night he had been suddenly seized with excruciating pain in the bowels, and although he appeared in great suffering, he said it had materially abated, his bowels had not acted for three days, and for some time previously but scantily and irregularly; his tongue was loaded with a thick yellow coating, with a strip down the centre of a brownish tint, and the breath tainted, with a fecal odor: the circulation but little disturbed. On examining the abdomen, the colon could be traced nearly its whole length with the eye; its ascending and transverse portion were loaded, except about two and half inches of the left side of the arch, and there induration and contraction of its coats were very perceptible; the centre of the induration for about half an inch was firmer and more contracted than the rest. We ought to have mentioned before, that he had felt a degree of uneasiness, scarcely amounting to pain, about the indurated portion for six or seven years, and at times had suffered severely from constipation; but never equally with the present attack. The descending portion of the colon was enormously distended; perhaps it would be more correct to say, to its utmost limit. His general health was considerably impaired, and he was more gloomy and desponding than ever. We gave him ten grains of hydrarg. chlorid., three of pulv. ipecac, and the sixth of a grain of morphine, to be followed in an hour with ol. ricini one ounce, spt. terebinth. half an ounce, and ordered the bowels to be well rubbed with the common volatile liniment; and prepared an injection of mucilage and turpentine, to be administered as soon as any effect from the medicine taken, was felt in the bowels. We saw him in about ten hours after, the medicine had acted thoroughly, and the colon was completely emptied. He was now ordered to take sufficient of the oil and turpentine daily to keep the bowels gently open, and to have the same injection administered every second or third day. This treatment was continued for a week when the medicine was omitted; the embrocation alone being continued. There was no perceptible alteration in the induration, nor was any expected. In a little more than two weeks we were again sent for in great haste, the messenger stating—"although he says he feels much better, I am of a different opinion, for some gathering has broken in his inside, and he has passed such strange looking matter from his bowels, that all who have seen it are convinced he must die." We found on our arrival that he had passed a considerable quantity of adipocere—amounting as nearly as we could judge, to some four or five ounces: this was passed out of doors. We directed him to use a vessel; and during a week he came to pass adipocere more or less in every stool—several chips of wood passed, some free, some imbedded in adipocere and bones, in the same connection, which were evidently those of a squirrel. We did not give any medicine, as nature was satisfactorily doing the work. From that time to the present, his health has been good, the induration is still there, but not near as firm or wide, but the contraction evidently does not interfere with the functions of the bowel. He is convinced that the last squirrel he eat was in Ohio, some seven or eight years before the attack, and the last chips that he had chewed and swallowed, were a little over three years before. I will merely add that he is one of our most intelligent citizens, and one whose word is never doubted by those who know him.

We shall be brief in our remarks on turpentine in typhoid-fever—reserving them for our communication to the committee on practical medicine, before the next meeting of the State Society. We will give only two short extracts from Dr. Copland's article on typhoid fever—and he has precedence of Dr. Wood by twelve years, and was, we believe, the first Physician who used turpentine in this form of fever. It perhaps may be well to state that at the time he wrote, the profession in England to a man believed in the idendity of typhus and typhoid fever. "If hemorrhage from the bowels occur, it may be ascribed chiefly to exudation from the softened mucous surface, as shown by post-mortem appearances." "I have likewise employed, by the mouth, and in enemata, the spirits of turpentine, which generally proves the most active remedy of any in such circumstances. In some hopeless cases it has succeeded contrary to expectations. If the disease be far advanced or the powers of life much reduced, the turpentine should be given in small or moderate doses, and its effects carefully watched (op. cit. page 1023), published in 1835." "The *Spirits of Turpentine* are frequently productive of benefit, when prescribed in small doses, with aromacits and spices; but a large dose may be attended by very serious consequences, when exhaustion is extreme. It is an excellent medicine in enemate, with castor-oil, chloride of sodium or other purgatives when the bowels require to be opened; or with assafœtida, or extract of rue, when there is much tympanitic distension, Substances of a similar kind, or the usual carminatives, have been directed in enemata by THOMAN and HUFELAND, in order to remove this symptom; but the injection just recommended is the most certain in its effects," (op. cit. page 1037.)

*(To be continued.)*

